# Effect of “DXB-2030,” a Polyherbal Formulation, on Experimental Polycystic Ovary Syndrome Associated with Hyperandrogenism

**DOI:** 10.1155/2019/8272850

**Published:** 2019-02-03

**Authors:** Mohammed Azeemuddin, Suryakanth D. Anturlikar, Mallappa Onkaramurthy, Mirza R. Baig, Basti K. Ashok, Raghavendra P. Rao, Mohamed Rafiq, Paramesh Rangesh

**Affiliations:** ^1^Discovery Sciences Group, R&D Center, The Himalaya Drug Company, Bangalore, Karnataka, India; ^2^Department of Microbiology & Toxicology R&D Center, The Himalaya Drug Company, Bangalore, Karnataka, India; ^3^Chief Scientific Officer, R&D Center, The Himalaya Drug Company, Bangalore, Karnataka, India

## Abstract

The objective of the present study was to evaluate “DXB-2030,” a polyherbal combination of *Trigonella foenum-graecum*, *Aloe vera*, *Sphaeranthus indicus*, *Nardostachys jatamansi*, and *Symplocos racemosa* extracts in an experimental model of testosterone propionate (TP), induced polycystic ovary syndrome (PCOS) in female rats. Thirty animals were divided into 3 groups of 10 each; group 1 served as normal control; group 2 was administered with TP and served as positive control; along with TP, group 3 was treated with “DXB-2030” at a dose of 100 mg/kg p.o., for 60 days. At the end of the study period, the animals were subjected for the estimation of serum testosterone levels, oral glucose tolerance test (OGTT), weight of the ovaries, estrous cycle, and histopathological evaluation. An in vitro assay on GLUT4 expression was carried out to understand the effect of “DXB-2030” on insulin resistance. Results showed that treatment with “DXB-2030” reversed the TP-induced changes by increasing the GLUT4 expression and decreasing the body weight, testosterone levels, AUC of glucose in OGTT, and the cystic follicles of the ovaries, thus indicating its beneficial effect in PCOS by ameliorating the metabolic dysfunction and reproductive impairment, which are the pathophysiological conditions associated with PCOS. From the results obtained, it can be concluded that “DXB-2030” was effective in the management of experimental PCOS and hence may be recommended in the treatment of PCOS.

## 1. Introduction

Polycystic ovary syndrome (PCOS) has become a major area of concern as it is affecting 12–21% of reproductive-aged women causing infertility [1]. This syndrome is characterized by multiple disorders such as hyperandrogenism, hyperinsulinemia, dyslipidemia, obesity, insulin resistance, anovulation, and cystic follicles in the ovary [2]. Almost 30–40% of women affected with PCOS have impaired glucose tolerance [3]. If early intervention is ignored, it may lead to other serious consequences such as type II diabetes mellitus (DM), cardiovascular disorders, and ovarian cancer [4]. According to the widely accepted Rotterdam Consensus, women can be diagnosed with PCOS when they exhibit at least any two of three features: androgen excess, ovulatory dysfunction, and polycystic ovary appearance on ultrasound after excluding other causes of these abnormalities [5].

Due to complex nature, complete understanding of pathogenesis of PCOS is still lacking. There are multiple pathophysiological mechanisms and various theories which have been proposed to explain the pathogenesis of PCOS. Some of the mechanisms are an alteration in gonadotropin releasing hormone (GnRH) secretion, increase of luteinizing hormone (LH) secretion, and alteration in insulin secretion which leads to hyperinsulinemia and insulin resistance (IR). Insulin resistance is one of the key players in the pathophysiology of PCOS. Insulin resistance and high insulin levels directly and indirectly stimulate ovarian theca cells to secrete androgens, and these result in an increase of androgen levels. The defect in androgen synthesis leads to increase in ovarian androgen production and ultimately to PCOS [6–8].

Currently, many of the treatments for PCOS primarily depend on desired clinical effect and include insulin sensitizers, infertility treatment, regulation of menstrual disturbances, antiandrogens, and hormonal therapies. But, all these treatments or treatment regimens have their own side effects. Either they are associated with substantial cost or may cause various side effects, such as irregular menstruation, gastrointestinal disturbances, weight gain, and increased insulin resistance [9–11]. The side effects of these medicines and their identification have significant importance in PCOS management. Many studies including randomized controlled trials, case studies, and animal experiments are focused on investigation of herbal drugs in this condition. Hence, exploration of potential herbs and their combination needs to be evaluated for the treatment or to have a check on all the aspects of pathogenesis of PCOS [11–13].

Based on the etiology and pathogenesis of PCOS, a polyherbal formulation “DXB-2030,” which is a combination of *Trigonella foenum-graecum*, *Aloe vera*, *Sphaeranthus indicus*, *Nardostachys jatamansi*, and *Symplocos racemosa* was prepared. The herbs are mixed in a right proportion as per the ayurvedic criteria to obtain a blend which can be targeted against multiple ramifications of PCOS in a holistic approach. The individual herbs used in this combination are reported to have significant relevance to the pathogenesis of PCOS. *Trigonella foenum-graecum* is used as insulin sensitizer in diabetes mellitus and also used in female reproductive disorders [14–17]. *Aloe vera* is known to bring estrus cyclicity to normalcy by controlling hyperglycemic conditions and modulating steroidogenesis and thus is a potential candidate for the maintenance of PCOS [18, 19]. *Sphaeranthus indicus* is reported to possess anxiolytic, central nervous system depressant and anticonvulsant activities, thus supporting its use in the management of anxiety related to PCOS [20, 21]. *Nardostachys jatamansi* is used in the management of stress causes due to various etiologies [22]. Research has shown its usefulness in the treatment of PCOS due to its antiandrogenic activity [23]. *Symplocos racemosa* is used in menorrhagia and other female reproductive dysfunctions which are some of the symptoms of PCOS [24, 25].

Some of the known actives identified in this combination “DXB-2030” are saponins, flavonoids, alkaloids, volatile oil, and polyphenols. The herbs used in this combination were carefully selected to balance the reproductive and metabolic aspects of PCOS. Based on the reported pharmacological properties of the herbs, the present study was designed to explore the effect of “DXB-2030” in the experimental model of PCOS in rats.

## 2. Materials and Methods

### 2.1. Chemicals

Testosterone propionate (TP), olive oil, and D glucose were obtained from HiMedia Laboratories Pvt. Ltd., Mumbai, India. Glucose oxidase kit was procured from Erba Mannheim (Transasia), Mumbai, India. Testosterone kit was purchased from Diagnostics Biochem Canada (DBC), Ontario, Canada.

### 2.2. Experimental Animals

Inhouse-bred female Wistar rats (9 days old) were housed in standard conditions of temperature (22 ± 3°C), relative humidity (55 ± 5%), and light (12 hr light/dark cycles) before and during the study. Animals were fed with standard pellet diet (Provimi Animal Nutrition India Pvt. Ltd.) and water *ad libitum*. The experimental protocol was approved by the Institutional Animal Ethics Committee (IAEC) of The Himalaya Drug Company, Bangalore, and the animals received humane care as per the guidelines prescribed by the Committee for the Purpose of Control and Supervision on Experiments on Animals (CPCSEA), The Ministry of Environment & Forests, Government of India.

### 2.3. In Vitro Studies

#### 2.3.1. GLUT4 Expression Study

*(1) Cell Culture and Treatment*. Differentiated C2C12 myotubes (1.2 × 10^5^ cells/well in a 6-well plate, procured from ATCC) were incubated with 500 *μ*M palmitate for 24 hours in DMEM high-glucose media. After the incubation, the cells were washed with sterile PBS and further incubated with the “DXB-2030” sample at nontoxic concentration of 200 *µ*g/ml (toxicity as determined from MTT assay) with and without 100 nM insulin in a 37°C incubator with 5% CO_2_ for 24 h. After incubation, media were removed, and the resulting adherent cells were subjected for total RNA isolation for further experimentation.

*(2) Gene Expression*. Total RNA was extracted from treated myotubes (*n* = 3) using RNA isolation kit (Krishgen Biosystem). The isolated RNA was quantified on agarose gel. Total RNA and random primers were used for the first-strand cDNA synthesis by reverse transcriptase. The PCR amplification was carried out in a reaction volume of 20 *µ*l containing 2 *µ*l of cDNA and 10 *µ*l of SYBR Green Supermix (BioRad, USA). The GLUT4 expression levels were normalized to that of 18s rRNA expression, and control was normalized to 1.

### 2.4. In Vivo Studies

#### 2.4.1. Testosterone Propionate-Induced PCOS

PCOS was induced in 20 animals and 10 remained as normal control. On the 9^th^ day of birth, testosterone propionate (TP) was administered at a dose of 1.25 mg/pup s.c. (1.25 mg TP in 0.02 ml of olive oil) to induce PCOS in 20 female pups, and olive oil 0.02 ml/pup was administered to 10 pups which served as normal controls. Pups were kept with respective mother until weaning; after that, they were housed in respective groups. After 70 days of age, vaginal smear was monitored daily to confirm the development of PCOS [26, 27].

Thirty animals were divided into 3 groups of ten each. Groups 1 and 2 were animals administered with demineralized water at a dose of 10 ml/kg b.wt. and served as normal and PCOS control, respectively, and group 3 was PCOS animals treated with “DXB-2030” at a dose of 100 mg/kg b.wt.p.o. for 60 days.

#### 2.4.2. Oral Glucose Tolerance Test (OGTT)

OGTT was performed one day before the terminal sacrifice. Glucose (2 g/kg) was administered to overnight-fasted rats to perform OGTT, and blood samples were collected from the retro-orbital plexus at 0 (before glucose load), 30, 60, and 120 min after glucose administration. Serum was separated, and serum glucose was estimated by the enzymatic glucose oxidase method.

#### 2.4.3. Estrous Cycle

Vaginal smear of all the animals were monitored daily in the last week of treatment and observed under microscope for the presence of different stages (proestrus, estrus, metestrus, and diestrus) of estrous cycle.

#### 2.4.4. Serum Testosterone Estimation

Two hours after the last dose of treatment, blood was collected from retro-orbital sinus under isoflurane anesthesia for the estimation of total testosterone (TT), and it was quantified using the ELISA method.

#### 2.4.5. Histopathological Evaluation

Briefly, after the blood collection, animals were euthanized using excess of anesthesia, and ovaries were excised, weighed, and fixed in 10% neutral buffered formalin and embedded into paraffin blocks. Tissue sections of 5 µm were cut and stained with hematoxylin and eosin and subjected for histopathological evaluation. The slides were evaluated using the microscope (Olympus, Nikon Eclipse E-400, Japan). The change in ovary like corpus luteum (CL), atretic follicle (AF), and cystic follicle (CF) was evaluated.

### 2.5. Statistical Analysis

All values are expressed as the mean ± standard error of the mean (SEM). The results were statistically analyzed by one-way analysis of variance (ANOVA) followed by Dunnett's comparison using Prism GraphPad 6.07 software, San Diego, CA, USA. A *p* value < 0.05 was considered statistically significant.

## 3. Results and Discussion

### 3.1. In Vitro Studies

#### 3.1.1. Effect of “DXB-2030” on GLUT4 Expression

**Figure 1:**
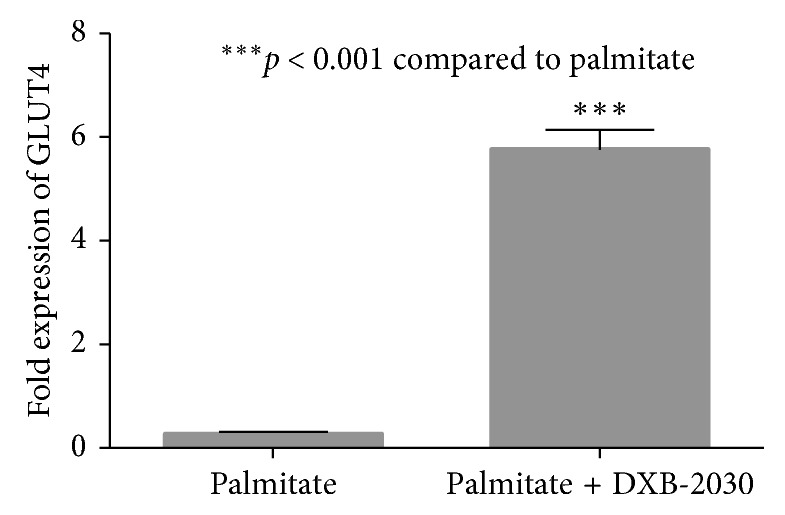
Effect of “DXB-2030” on GLUT4 gene expression: cells were treated with insulin under indicated conditions, total RNA was isolated, and the expression level of GLUT4 was analyzed by quantitative real-time PCR, using 18s RNA as internal control. Expression levels in control were normalized to 1. The level of significance is denoted as ^*∗∗∗*^*p* < 0.001 compared to the palmitate group. The unpaired *t*-test was used for statistical comparison.

Palmitic acid is known to induce the insulin-resistance condition in the myotubes which results in decreased glucose uptake. In our assay (which measures the GLUT4 expression levels), it was observed that the GLUT4 levels were very low even in the presence of insulin. When these cells were treated with “DXB-2030,” it resulted in increased expression of GLUT4 levels indicating an increased glucose uptake and hence increased insulin sensitivity (Figure 1).

### 3.2. In Vivo Studies

After 70 days of age, vaginal smear was monitored daily for 10 consecutive days, and the animals which exhibited irregular estrous cycle were considered as PCOS-positive animals and used for the study. All the animals administered with TP showed irregular estrus cycle and used for further evaluation.

#### 3.2.1. “DXB-2030” Reduces Body Weight

**Figure 2:**
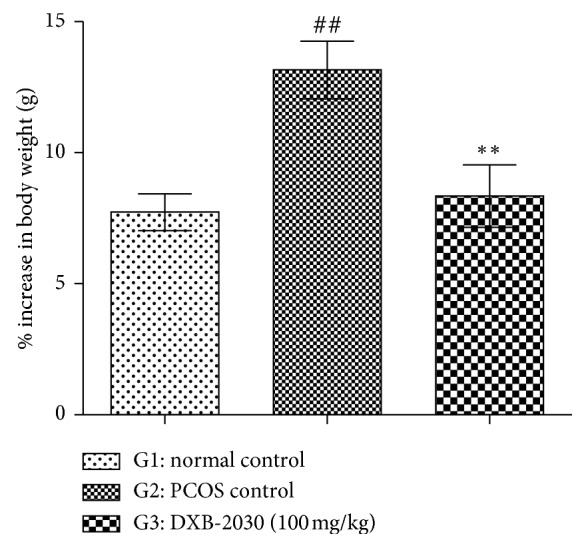
Effect of DXB-2030 on the percentage increase in body weight of rats: a significant increase in % body weight in PCOS control rats (^##^*p* < 0.01) compared to normal controls and significant decrease in % body weight in “DXB-2030” treatment group (^*∗∗*^*p* < 0.01) compared to PCOS controls were observed. One-way ANOVA was used for statistical comparison.

All animals were weighed weekly once in the study period till the end of the study. The percentage increase in the body weight was mentioned in the data. The data represent the increase in percentage body weight on the last day of treatment. An overall increase in body weight in all the group of animals over the experimental period was observed. A significant increase in the body weight was observed in the PCOS control group compared to the normal control group, whereas treatment with “DXB-2030” showed a significant decrease in body weight compared to the PCOS control group (Figure 2).

#### 3.2.2. “DXB-2030” Ameliorates Glucose Intolerance

**Figure 3:**
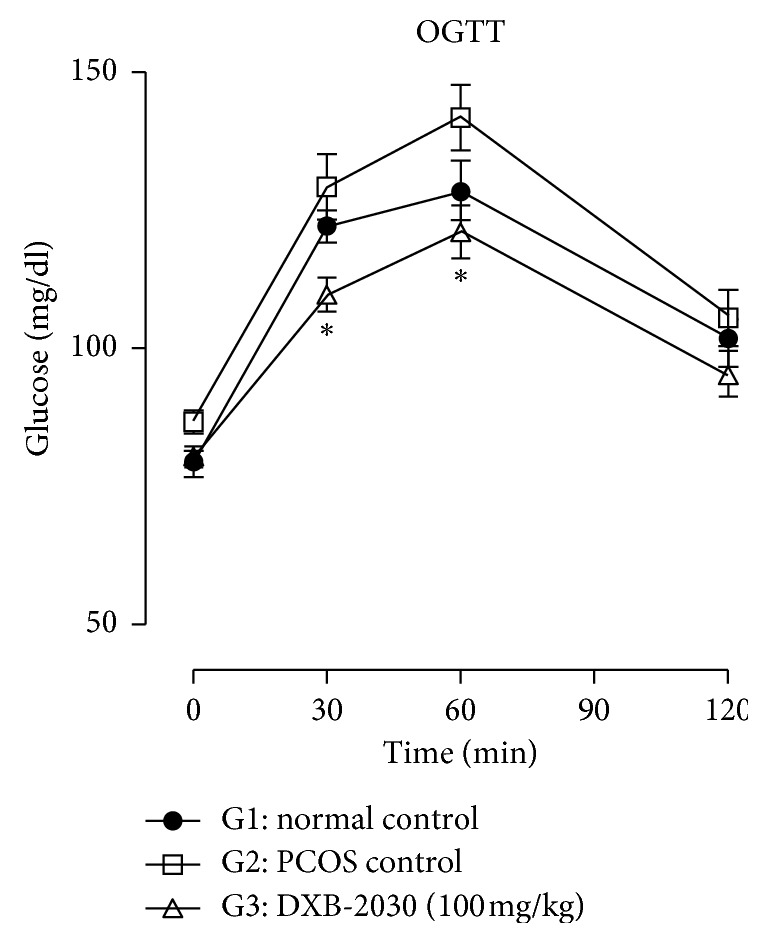
Effect of “DXB-2030” on the oral glucose tolerance test (OGTT) of rats: the glucose levels were estimated at 0, 30, 60, and 120 minutes. The “DXB-2030-treated” group showed a significant decrease in glucose levels at 30 (^*∗*^*p* < 0.05) and 60 (^*∗*^*p* < 0.05) minutes compared to the PCOS control group. One-way ANOVA was used for statistical comparison.

In the last week of the treatment, OGTT was performed, and intragastric administration of glucose did not produce many changes in normal control and showed the normal profile to glucose tolerance, whereas the PCOS control animals showed the increase in the glucose intolerance when compared to normal control. Blood glucose levels at 30, 60, and 120 min were higher in the PCOS group compared to the control group. Further “DXB-2030-treated” animals showed significant reduction in glucose levels at different time points over the period of 120 min when compared to the PCOS control group (Figure 3).

#### 3.2.3. “DXB-2030” Decreases Ovary Weight

**Figure 4:**
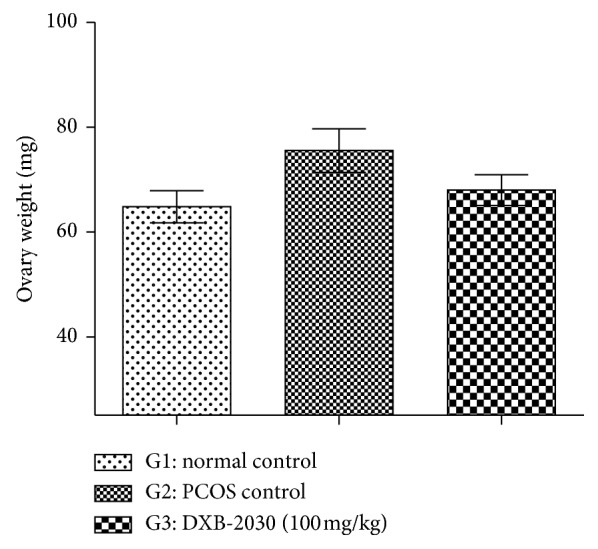
Effect of “DXB-2030” on ovary weight of rats: an increase in ovary weight in PCOS control rats and a decrease in ovary weight in “DXB-2030” rats were observed. The changes in ovary weight were not found to be statistically significant.

TP injection showed bilateral polycystic ovaries, increase in the ovary weight, and irregularity in the estrus cycle when compared to normal control, whereas treatment with “DXB-2030” showed decrease in the ovary weight and normalization of irregular estrus cycle (data not shown), when compared with the PCOS control group (Figure 4).

#### 3.2.4. “DXB-2030” Decreases Serum Testosterone Level

**Figure 5:**
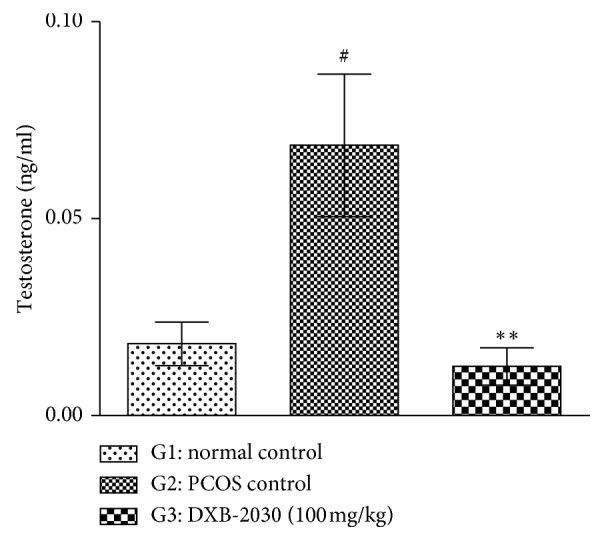
Effect of “DXB-2030” on serum testosterone of rats: a significant increase in serum testosterone levels in PCOS control group (^#^*p* < 0.05) and significant decrease in serum testosterone in the “DXB-2030” treatment group (^*∗∗*^*p* < 0.01) compared to PCOS control were observed. One-way ANOVA was used for statistical comparison.

The serum testosterone level was quantified by ELISA assay. Significant increase in the testosterone level of the PCOS control group was observed compared to the normal control group. Treatment with “DXB-2030” significantly suppressed the elevation of the testosterone level compared to the PCOS control group (Figure 5).

#### 3.2.5. Histopathological Changes in “DXB-2030” Treated Rats

The histoarchitecture of ovaries got disrupted due to the administration of TP. Animals showed an increase in cystic follicle and atrophic changes in the PCOS control group compared to normal control. Treatment with “DXB-2030” showed decrease in cystic follicle formation and atrophic changes when compared to PCOS control and an overall recovery in the histoarchitecture of ovaries was recorded (Table 1).

PCOS is a heterogeneous disorder linked with both reproductive and metabolic dysfunction. The etiology of PCOS is complex and multifactorial. Women with PCOS are usually diagnosed with irregular menstrual cycles, altered hormone levels, and also presence of ovarian cysts [28]. Further evidence also suggests that overweight or obesity with decreased glucose tolerance is a common feature of metabolic dysfunction which plays an important role in development of PCOS [29]. Multiple mechanisms are involved in the pathogenesis of the PCOS. Mainly polycystic ovaries develop when the ovaries are stimulated to produce a large amount of male hormones/androgens mainly testosterone. This stimulation may be due to the release of excessive luteinizing hormone (LH) by the anterior pituitary gland or by high levels of insulin in the blood of women whose ovaries are sensitive to this stimulus or due to reduced levels of sex hormone-binding globulin (SHBG) resulting in increased free androgens [30]. In some cases, women with PCOS will have total testosterone levels within the normal range but will be clinically hyperandrogenic; this is due to elevated free testosterone levels [31].

Insulin regulates the glucose homeostasis by enhancing the glucose uptake by muscle and adipose tissue while suppressing the glucose output by the liver cells. C2C12 myotubes when incubated in the presence of insulin show increased glucose uptake, and this can be indirectly measured by the level of expression of GLUT4. This assay is employed to measure the insulin sensitivity [32]. In this study, the C2C12 myotubes were employed to study the effect of different phytoactives on insulin-mediated glucose uptake. GLUT4 expression levels were measured by qPCR as a surrogate for glucose uptake. Various combinations were subjected for this assay, and based on the outcome and the Ayurvedic wisdom, “DXB-2030” was finalized for further evaluation.

Based on the understanding of pathophysiology mentioned above, the experimental model of TP-induced PCOS was selected to evaluate “DXB-2030” for its beneficial effect in PCOS. This model was found to interfere with the reproductive and metabolic function of the female rats. It causes a change in normal morphology of the reproductive tract and disturbance in the duration of the particular phase of the estrous cycle. The changes in the estrous cycle, hyperandrogenism, hormonal imbalance, and presence of peripheral cysts in the ovaries due to TP administration are some of the symptoms comparable to reproductive anomalies of human PCOS [33, 34].

“DXB-2030” is prepared based on the Ayurvedic relevance, published literature, and in vitro efficacy studies performed on the use of individual herbs in the various symptoms of PCOS. These herbs have shown the activity on reproductive disorders, improved glycemic control, and decrease in insulin resistance, androgen receptor inhibition and anxiolytic effect. The individual herbs present in this combination are reported of exerting their beneficial effects on the female reproductive system. *Trigonella foenum-graecum* seed extract showed encouraging results in 94% of patients, and surprisingly, 12% of study population got pregnant and showed significant improvement in regulating the menstrual cycle [35]. In another clinical study, *Trigonella foenum-graecum* seed extract showed significant reduction in ovary volume and size of the cyst. It also showed significant increase in luteinizing hormone (LH) and follicular stimulating hormone (FSH) levels compared to the baseline values [17]. *Aloe vera* leads to reversion of estrus cyclicity to normal by controlling hyperglycemic conditions and modulating steroidogenesis, and thus, it is the potential candidate for the maintenance of PCOS, which was supported by many preclinical studies [18, 19]. *Sphaeranthus indicus* is reported to be used in the management of anxiety and stress related to PCOS [20, 21]. *Nardostachys jatamansi* showed its usefulness in the treatment of PCOS by its antiandrogenic effect [22, 23]. *Symplocos racemosa* bark is given in menorrhagia and other female reproductive dysfunctions which are some of the symptoms of PCOS. Experimental studies show that *S. racemosa* treatment significantly decreased the elevated testosterone levels and restored estrogen, progesterone, and cholesterol levels. It also restored the normal weight and histology of ovarian tissue. These effects of *S. racemosa* were found to be comparable with clomiphene citrate [24, 25].

The possible mechanism of “DXB-2030” may be due to the inhibition of androgen receptors which aggravated due to the administration of TP, which further reduces the testosterone concentration which is responsible for the development of PCOS. The metabolic dysfunction which is associated with PCOS due to glucose intolerance and decreased glucose uptake was corrected with the treatment of “DXB-2030” may be by the upregulation of GLUT4 expression and increasing glucose tolerance, thus increasing the insulin sensitivity.

## 4. Conclusion

Intervention with “DXB-2030” reverses the pathophysiological changes caused due to the administration of TP in immature female rats. The beneficial effect of “DXB-2030” on PCOS may be due to the synergistic effect of the individual herbs which are known to exert their effect on the abnormal female reproductive system by various mechanisms like, reversion of estrus cyclicity, reduction in ovary volume and size of the cyst, antiandrogenic effect, decreased testosterone levels and restoration of the histology of ovarian tissue. Based on the outcome of the study, it can be inferred “DXB-2030” was found to be useful in the treatment of PCOS. However, further experimental and clinical studies are required to confirm the same and to derive the exact mechanism of action of “DXB-2030.”

## Figures and Tables

**Table 1:** Histopathological evaluation of ovaries in testosterone propionate-induced PCOS rats: increase in cystic follicles and atrophic changes were observed in PCOS control rats, and these changes were reversed with the treatment of “DXB-2030.”

Groups	Corpus luteum (%)	Cystic follicle (%)	Atrophic changes (%)
G1: normal control	95	10	0
G2: PCOS control	70	75	80
G3: DXB-2030 (100 mg/kg b.wt.)	78	63	57

## Data Availability

The experimental data used to support the findings of this study are included within the article.
